# The pentacyclic triterpenoid, plectranthoic acid, a novel activator of AMPK induces apoptotic death in prostate cancer cells

**DOI:** 10.18632/oncotarget.6625

**Published:** 2015-12-16

**Authors:** Nosheen Akhtar, Deeba N. Syed, Mohammad Imran Khan, Vaqar M. Adhami, Bushra Mirza, Hasan Mukhtar

**Affiliations:** ^1^ Department of Dermatology, School of Medicine and Public Health, University of Wisconsin, Madison, WI 53706, USA; ^2^ Department of Biochemistry, Faculty of Biological Sciences, Quaid-i-Azam University Islamabad, Islamabad 45320, Pakistan

**Keywords:** 5′AMP-activated kinase (AMPK), plectranthoic acid, prostate cancer (PCa)

## Abstract

Epidemiologic studies indicated that diabetics treated with metformin had a lower incidence of cancer than those taking other anti-diabetes drugs. This led to a surge in the efforts for identification of safer and more effective metformin mimetic compounds. The plant *Ficus microcarpa* is widely used for the treatment of type 2 diabetes in traditional medicine in South Asia. We obtained extracts from this plant and identified a small molecule, plectranthoic acid (PA), with potent 5′AMP-activated kinase (AMPK) activating properties far superior than metformin. AMPK is the central hub of metabolic regulation and a well-studied therapeutic target for metabolic syndrome, type-2 diabetes and cancer. We observed that treatment of prostate cancer (PCa) cells with PA inhibited proliferation and induced G0/G1 phase cell cycle arrest that was associated with up-regulation of cyclin kinase inhibitors p21/CIP1 and p27/KIP1. PA treatment suppressed mTOR/S6K signaling and induced apoptosis in PCa cells in an AMPK-dependent manner. Interestingly, PA-induced autophagy in PCa cells was found to be independent of AMPK activation. Combination studies of PA and metformin demonstrated that metformin had an inhibitory effect on PA-induced AMPK activation and suppressed PA-mediated apoptosis. Given the anti-proliferative role of PA in cancer and its potent anti-hyperglycemic activity, we suggest that PA should be explored further as a novel activator of AMPK for its ultimate use for the prevention of cancers and treatment of type 2 diabetes.

## INTRODUCTION

5′-AMP-activated protein kinase (AMPK) is a key target in maintaining energy homeostasis. It is heterotrimeric serine/threonine kinase, consisting of a catalytic α subunit and two regulatory subunits (β and γ). AMPK can be activated by extracellular changes, such as, depletion of ATP, low glucose, and changes in NADPH levels [1]. Activation of AMPK stimulates catabolic pathways leading to ATP production such as fatty-acid oxidation and blocks anabolic processes that consume ATP such as lipid and cholesterol synthesis, increases glucose uptake and stimulates autophagy [2]. The role of AMPK in regulating metabolism is well understood; predominately studied in the context of type-2 diabetes and metabolic syndrome. More recently, studies have shown that AMPK is a possible metabolic tumor suppressor and target for cancer prevention and possibly cancer treatment [3]. Reports are available for the role of AMPK in melanoma, leukemia, ovarian, prostate and breast cancers [4–8]. AMPK inhibits/regulates mTOR which is consistently deregulated in cancer cells, by activating TSC1/TSC2, which results in dephosphorylation of the translation inhibitor 4E-BP1 and the ribosomal protein S6 kinase (S6K), consequently leading to termination of protein translation. Thus, AMPK plays a central role in the control of cell growth, proliferation and autophagy. Furthermore, AMPK directly phosphorylates p53 on S^15^ residue, which is essential for G1-phase cell cycle arrest [9]. Accordingly, in recent years it is increasingly appreciated that AMPK activators may be a suitable therapeutic target against human cancers.

Studies have shown that activated AMPK is expressed frequently in primary human prostate cancer specimens of various Gleason grades, suggesting its involvement in the growth and survival of human prostate cancer cells. Park *et al*. have further reported that the growth of human prostate cancer cells is AMPK-dependent and its inhibition suppresses cell proliferation, suggesting its potential as a novel target for prevention and treatment of human prostate cancer [10]. In contrast, Kim et al, exploring the mechanistic basis of Remotiflori radix ethanol extract (ERR)-induced death in prostate cancer cells demonstrated that knockdown of AMPK dramatically blocked ERR-mediated apoptosis [11]. However, more recent studies have identified the energy sensor AMPK as a viable therapeutic target in prostate cancer. Zadra *et al*. showed that the AMPK activator MT 63-78 was a potent inhibitor of prostate cancer cell growth in androgen sensitive and castration resistant models, resulting in mitotic arrest and apoptosis [12].

Many compounds activate AMPK such as 5, amino-imidazol-4-carboxamide-1-b-4-ribofuranoside (AICAR), metformin, phenformin, buformin and some non-steroidal anti-inflammatory drugs (NSAIDs) [13, 14]. Natural flavonoids (quercetin), polyphenols (resveratrol), anthocyanin and berberine have also been shown to activate AMPK [15, 16]. Although several AMPK stimulators have been identified, each has its own limitations. AICAR has a short half-life and poor bioavailability, phenformin and buformin induce lactic acidosis, and metformin may increase the risk of death in non-obese individuals. These activators are also known to have additional molecular targets and AMPK-independent toxic effects which reduce their efficacy in cancer [17–19]. In summary, there is a need to develop novel therapeutic compounds that target this multifunctional protein kinase at low concentrations [20, 21]. In addition, most AMPK stimulators at low concentration are effect at very high concentrations, posing the possibility of undesired effects.

We isolated a novel triterpenoid plectranthoic acid (PA) from *Ficus microcarpa*, a traditional plant with antidiabetic properties. Our studies exploring its anti-hyperglycemic effects showed that PA inhibited amylase, glucosidase and DPP4 activities suggestive of its possible role in the treatment of type 2 diabetes (unpublished data). The purpose of the current study was to study the effects of PA, using prostate cancer cells and investigate whether AMPK serves as its molecular target in mediating the observed anti-proliferative effects. We observed that PA is a potent activator of AMPK with therapeutic potential against prostate cancer.

## RESULTS

### Crude extract of *Ficus microcarpa*, its fractions and isolated compounds inhibit proliferation of cancer cells

**Figure 1:**
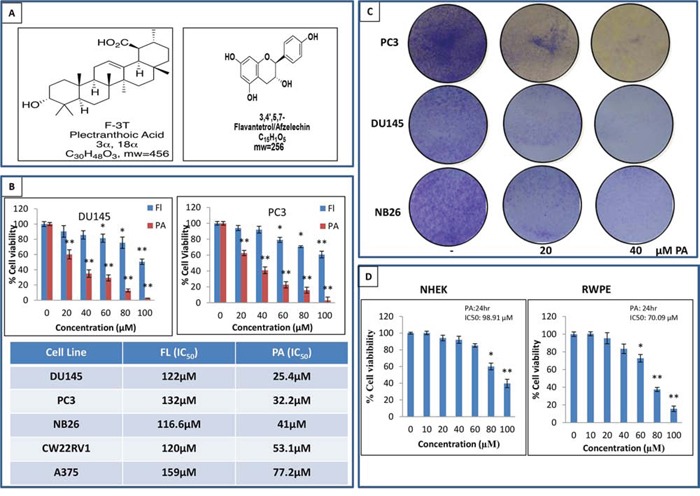
PA inhibits cancer cell proliferation and is non-toxic to normal cells **A.** NMR identified structure of PA and FL. **B.** Effects of PA and FL on the viability of melanoma and prostate cancer cells. Cells were treated with PA and FL at the indicated concentrations for 24h, and cell viability was assessed by MTT assay. Table shows the IC_50_ of PC3, DU145, CWRV1, NB26 and A375 cells at 24h. Mean ± SD of experiments performed in triplicate is shown. **C.** Dose-dependent effect of PA on clonogenecity of PC3, DU145 and NB26 cells as detected by colony formation assay. Details are described in material methods. **D.** Effect of various concentrations of PA on viability of normal cells i.e. RWPE and NHEK, as determined by MTT assay.

Through liquid-liquid fractionation three fractions, n-hexane (FMN), ethyl acetate (FME) and aqueous (FMA) were prepared from crude extract of *Ficus microcarpa* (FMC). FME showed toxicity to *Agrobacterium tumefaciens* virulent strains At10 in potato disc tumor assay (Sup. Figure 1). Two pure compounds were isolated from FME via column chromatography which were then characterized through NMR and identified as PA and 3, 4, 5 flavantetrol (FL) (Sup. Figure 2). The structure of both compounds is shown in Figure 1A.

The crude extract and fractions were evaluated for their efficacy in inhibiting the viability of cancer cells. Employing the 3-(4, 5-dimethythiazol-2-yl)-2, 5-diphenyl tetrazolium bromide (MTT) assay, we initially evaluated the anti-proliferative activity of the crude extract and its fractions in melanoma (A375) and prostate (DU145, PC3, CWRV1 and NB26) cancer cells. Results showed that FMC, FMN, FME and FMA treatment (10-100μg/ml:24 h) inhibited the growth of cancer cells in a dose dependent manner. However, FME was found to be more potent than other fractions in the various cell lines examined (Sup. Figure 3A). Next, we evaluated the anti-proliferative activity of the isolated compounds (PA and Fl) at 24h. PA significantly inhibited the viability of DU145, PC3, CWRV1, NB26 and A375 cells with IC_50_ values ranging from 25.4, 32.2, 41, 53.1 to 77μM, respectively (Figure 1B). As time course analysis revealed only a modest difference between IC_50_ values of PA at 24h and 48h (Sup. Figure 3B) further studies were performed at the 24h time point. Antiproliferative effect of PA was further assessed by BrDU assay on DU145, PC3 and NB26 prostate cancer cells and results confirmed its anti-proliferative activity (IC_50_: 35, 42, 61μM respectively) (Sup. Figure 4). Clonogenic assays validated these findings, where selected concentrations 20μM and 40μM showed a significant dose-dependent inhibition of colony formation relative to untreated controls (Figure 1C). Finally to ascertain if PA was toxic to normal cells we performed MTT assay on prostate epithelial (RWPE) cells and human epithelial keratinocytes (NHEK). The IC_50_ values of 70.09μM and 98.91μM for RWPE and NHEKs indicated that PA had no effect on the growth of normal cells at doses which inhibited proliferation of cancer cells (Figure 1D).

### PA induces G0/G1 phase arrest in prostate cancer cells

**Figure 2:**
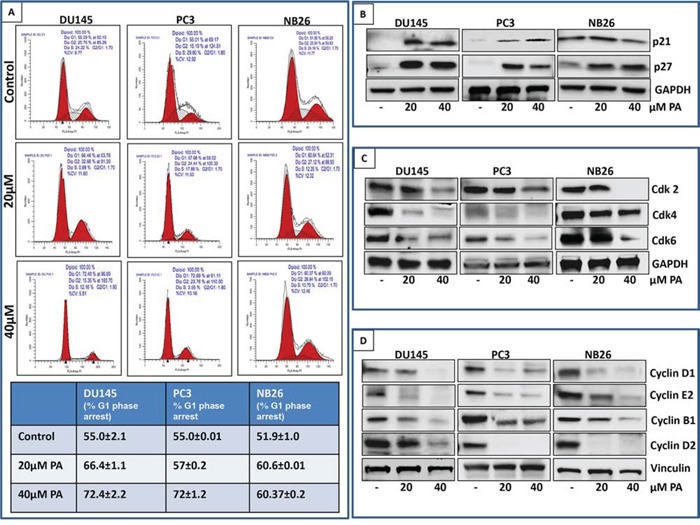
PA induces G0/G1 phase arrest of prostate cancer cells **A.** PC3, DU145, and NB26 were treated with 20-40μM PA for 24h, fixed with 1% paraformaldehyde and cell cycle distribution was evaluated after propidium iodide staining. Cells were analyzed by flow cytometry. Percentage of cell population in G0/G1-phase of the cell cycle is shown in the table. Experiments were performed in triplicate and mean ± SD is shown. **B, C & D.** Effect of PA treatment on cell cycle regulatory proteins: Whole cell lysates of PC3, DU145 and NB26 cells with/without PA (20–40μM:24h) were subjected to SDS-polyacrylamide gel electrophoresis. Equal loading was confirmed by reprobing with GAPDH. The immunoblots shown are representative of three independent experiments with similar results.

We next evaluated the cell cycle profile of prostate cancer cells treated with PA. Cells (DU145, PC3 and NB26) were treated with PA (20μM&40μM:24 h) and cell cycle analysis was performed using flow cytometry. Results showed that 24h treatment with PA induced significant enrichment in the G0/G1 phase with remarkable decrease in the fraction of cells in S phase (Figure 2A). Our data showed that 66.46% (20μM PA) and 72.4% (40μM PA) cells were arrested in G0/G1 phase in DU145, 57.68% (20μM) and 72.69% (40μM) in PC3 and 60% (20&40μM) in NB26 as compared to untreated controls (55.03%, 55.01% and 51.90%, respectively). There was a corresponding decrease in number of cells in S phase (0.88%: 12.16% vs 24.22% for control in DU145, 17.88%: 3.55% vs 29.8% control in PC3, 12.25%: 10.7% vs 24.16% for control in NB26). Western blot analysis was performed to determine the effect of PA treatment on cell cycle related proteins. A dose dependent decrease in cyclins (Cyclin B1, D1, D2 and E2) and Cdks (Cdk 2, Cdk4 and Cdk6), accompanied with induction of p21 and p27 was observed in PA treated cells (Figure 2B, 2C, 2D).

### PA induces apoptosis in prostate cancer cells

**Figure 3:**
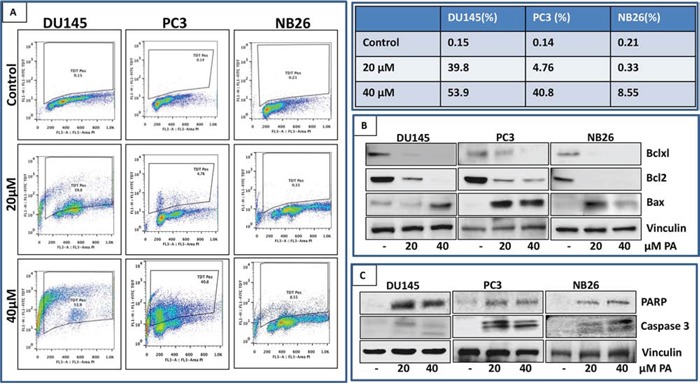
PA induces apoptosis in prostate cancer cells **A.** PC3, DU145 and NB26 cells treated with PA (20–40μM:24h) were labeled with FITC and analyzed by flow cytometry. Percentage of apoptotic cells with the corresponding dose of PA is shown in the table (*right*). **B&C.** Effect of PA treatment on proteins involved in apoptosis was evaluated. Whole cell lysates of PC3, NB26 and DU145 cells with/without PA (20–40μM:24h) treatment were subjected to SDS-polyacrylamide gel electrophoresis. Equal loading was confirmed by reprobing with GAPDH. The immunoblots shown are representative of three independent experiments with similar results.

After establishing through a series of experiments that PA is a potent antiproliferative compound, we next examined whether its growth inhibitory effect is associated with induction of apoptosis. For this purpose FITC staining of PA treated prostate cancer cells (DU145, PC3 and NB26) was performed out and analyzed by flow cytometry. There was a significant dose-dependent increase in apoptotic cell population in PA treated cells. As shown in Figure 3A, DU145 were more susceptible to PA with higher percentage of TdT positive cells (39.8% and 53.9%) as compared to PC3 (4.76% and 40.8%) and NB26 (0.33% and 8.55%)). Western blot analysis showed decreased expression levels of anti-apoptotic proteins such as Bcl2 and Bcl-xl in a dose dependent manner accompanied by an increase in the expression of pro-apoptotic protein Bax (Figure 3B). Induction of late apoptotic events was reflected by activation of terminal caspase-3 and detection of cleaved PARP (Figure 3C).

### PA is a potent activator of AMPK

**Figure 4:**
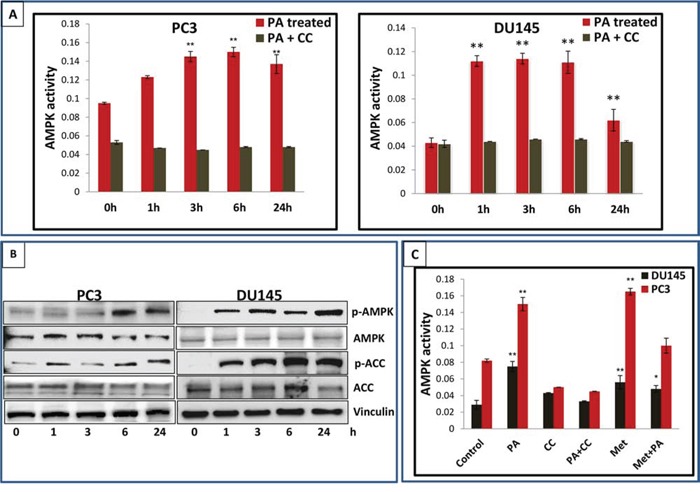
PA is a potent activator of AMPK **A.** PC3 and DU145 cells treated with PA (20μM) were harvested at different time points and AMPK activity assay was performed. **B.** Western blot analysis of the time-dependent effects of PA on the phosphorylation of AMPK and ACC. **C.** AMPK activity in PC3 and DU145 cells treated with PA (20μM), metformin 10mM and PA+ metformin. The results are expressed as normalized average ± S.D. of three independent experiments.

*In vitro* cell-free enzymatic assays indicated that PA possessed potent anti-hyperglycemic activity (unpublished data). Since AMPK has been identified as a target for anti-diabetic drugs, we asked whether PA modulates the activity of the kinase. For this purpose DU145 and PC3 cells were treated with PA (20μM) and incubated for different time periods (1, 3, 6 and 24h). AMPK activity assay revealed a robust increase in AMPK activity after PA treatment, in both cell lines (Figure 4A). Western blot analysis of whole cell lysates showed an increase in phosphorylation of AMPK α subunit at the Thr^172^ residue (Figure 4B). This was associated with phosphorylation of Acetyl CoA Carboxylase (ACC), in a time-dependent manner. Dose-dependent studies further confirmed the potential of PA as a potent AMPK activator (Sup. Figure 5). We next performed comparative studies to explore the effect of PA and metformin on AMPK activity in DU145 and PC3 cell lines. Our data showed that PA activated AMPK at 20μM while metformin mediated activation of AMPK occurred at 10mM. Interestingly, treatment of cells with a combination of PA and metformin resulted in a decrease in AMPK activity (Figure 4C).

### PA binds to γ subunit of AMPK with high affinity

**Figure 5:**
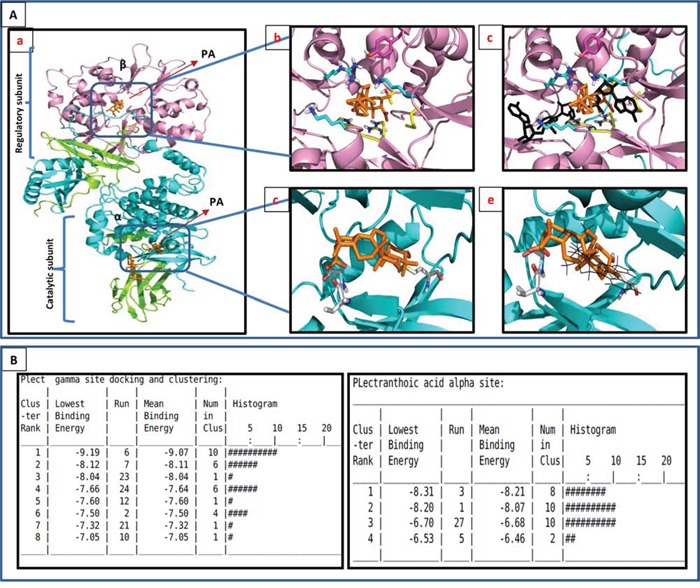
In-silico modeling of PA with AMPK **A.** PA docked to AMPK by autodoc4. The model used was the AMPK structure with ligands in the PDB entry 4CFF (DOI: 10.1038/ncomms4017, Nature Communications). α is cyan, β is green, γ is magenta and the ligands are gold. **Ab.** Docking results for the allosteric regulatory γ site. The ligand docks in a highly positive charged site. The nearest residues to the ligand are: Tyr^121^ (magenta), Arg^70^, Thr^81^, Met^85^, His^151^, Arg^152^. The carboxylate of PA is buried and interacts with Arg^152^. **Ac.** PA binds to the same regulatory site at which AMP (black) binds. **Ad.** Docking results for the α (kinase) binding site. The nearest residue are the backbone of Val^24^ at the carboxyl end, and Met^93^ at the -OH end. The primary interaction is hydrophobic. **Ae.** PA interferes with the binding site of staurosporine. **B.** Clustering data of PA to both α and γ subunits of AMPK.

We next determined the interaction of PA and AMPK by *in silico* docking. Blind docking was performed with Autodock4 (AD4 Scripps Institute) by preparing a docking volume that included a large amount of the protein surface. This allowed the docking program to examine many of the various surface features. Blind docking volumes were created for each of the three major domains in AMPK (Figure 5A). Each Autodock run produced 30 orientations of the docked molecules ranked on binding energy. The more negative energies represent the summation of physical terms that present the free energy of binding. The lower this energy, the more favorable is the docking orientation. Some of the orientations will have similar poses with similar energies and are considered to be a cluster of hits. Among α, β, and γ subunits, PA bound to γ subunit with high affinity (Figure 5Aa). The lowest, most preferred free energy of binding conformations within the top cluster at γ, α and β were −9.91Kcal/mol, −8.31Kcal/mol and −6.66Kcal/mol, respectively.

The γ site contains three AMP binding sites that AD4 selected as potential PA binding sites (Figure 5Ab). The most favorable site is an AMP binding surface pocket containing lysine (Lys^126^) and arginine (Arg^223^) residues (site A). In the most favorable orientation in site A, the carboxylate of PA hydrogen bonds to these residues and spans the pocket to bind the hydroxyl of PA to the backbone of the protein in the bottom of the pocket (Figure 5Ac; Sup. Figure 6A). In an alternate cluster of nearly equal minimum energy (site B), the carboxylate and hydroxyl of PA flip positions, and the carboxylate is buried in the pocket. There are 10 orientations is the top cluster of site A, with a mean binding energy of −9.07 Kcal/mol. The inverse orientation cluster, site B, contains 6 poses with a mean binding energy of −8.11 Kcal/mol. Since AD4 is accurate to + 2 Kcal/mol, these two orientations are of equal probability.

Figure 5Ad shows the preferred binding mode in the α domain, the ATP binding domain. The carboxylate of the acid end of PA is hydrogen bonded to the backbone near the n-terminal domain while the hydroxyl on the opposite end of PA is buried in the kinase pocket (Sup. Figure 6B). This docking does not include the hydrogen bonding to the adenine binding loop found in common kinase inhibitors, and docks poorly compared to staurosporine (binding energy −9.2 Kcal/mol) (Figure 5Ae). The highly hydrophobic core of PA is interfaced with hydrophobic residues deep in the pocket. The top binding cluster contains 8 poses with a mean binding energy of −8.21 Kcal/mol (Figure 5B).

The β site was found to be an activator site involving the interface of α and β (Structural basis for AMP binding to mammalian AMP-activated protein kinase. In this structure, the carbohydrate binding domain, or the β domain, and theα domain are linked by a small molecule activator. This site contains the phosphorylated serine that is important in AMPK activation. Docking in this site with AD4 results in the weakest binding (Figure 5Aa). The carboxylate of PA is hydrogen bonding to the phosphate of the serine (Ser^108^) in the β domain while hydroxyl accepts a proton from the backbone of Asn^48^ in the α domain. The top orientation contains 11 in the cluster with a mean binding energy of −6.62 Kcal/mol.

### PA inhibits phosphorylation of mTOR and induces autophagy in prostate cancer cells

**Figure 6:**
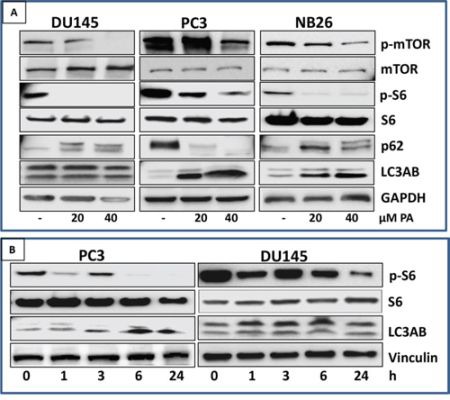
PA inhibits mTOR signaling and induces autophagy in prostate cancer cells **A.** PC3, NB26 and DU145 cells were treated with PA (20-40μM) for 24h. Cell lysates were subjected to western blot analysis for dose-dependent effects of PA on mTOR/S6K signaling. **B.** PC3 and DU145 cells were treated with PA (20μM) for 1h, 3h, 6h and 24h. Cell lysates were subjected to western blot analysis for time-dependent effects of PA on mTOR/S6K signaling Equal loading was confirmed by reprobing with GAPDH. The immunoblots shown are representative of three independent experiments with similar results.

We next turned our attention to pathways downstream of AMPK, known to play a vital role in the control of cell growth/proliferation and survival. Prostate cancer cells were treated with PA (20 μM) and the effect on mTOR signaling pathway and induction of autophagy was evaluated. Western blot analysis of PA treated PC, DU145 and NB26 cells showed remarkable decrease in phosphorylation of mTOR at Ser^2448^ and S6 Ser^236/237^ residues (Figure 6A). Next, distribution of the autophagy marker LC3 was assessed in these cells. PA treatment caused the induction of autophagy marker (LC3AB) in PC3 and NB26 but not in DU145 cells (Figure 6A). A time course analysis of S6K expression was conducted, as readout for mTOR signaling. PA treated cells showed an initial reduction in phosphorylation of S6 at 1h followed by an increase at 3h, after which there was a significant reduction in phosphorylation at all time points studied. Moreover, induction of autophagy correlated to conversion of LC3-I to LC3-II (LC3B) was observed at 6h which persisted till 24h in PC3 cells. Remarkably, there was no induction of LC3B at any time point in DU145 cells (Figure 6B).

### Inhibition of mTOR signaling and induction of apoptosis by PA is dependent on AMPK activation

**Figure 7:**
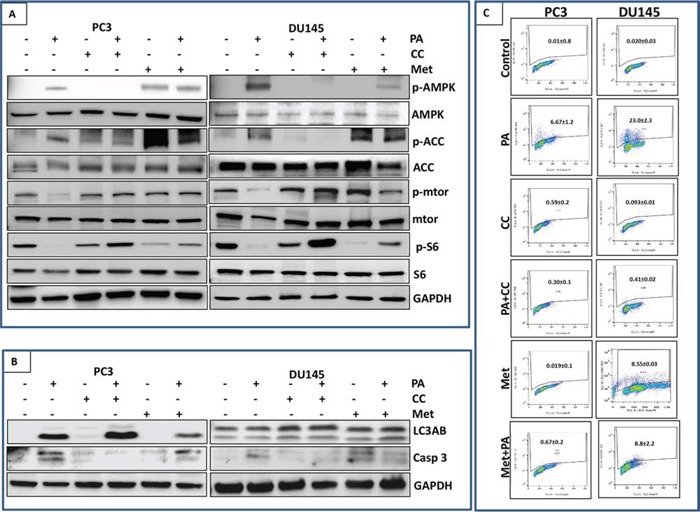
Inhibition of mTOR signaling and induction of apoptosis by PA is dependent on AMPK activation **A.** PC3 and DU145 cells were treated with PA (20μM) with or without compound C (20μM), metformin (10mM) and metformin (10mM) with PA (20μM) for 24h. Cell lysates were then examined for mTOR signaling downstream of AMPK using western blotting. **B.** Cells lysates of PC3 and DU145 were analyzed for expression of autophagy marker (LC3AB) and apoptosis marker (caspase 3) after treating with PA alone or in combination with compound C and metformin. **C.** Cells were labeled with FITC and analyzed by flow cytometry. Percentage of apoptotic cells with the corresponding dose of PA or CC is shown on the histograms. The experiment was performed in triplicates and repeated thrice.

Based on the results above, we hypothesized that inhibition of mTOR and induction of autophagy and apoptosis by PA is dependent on AMPK activation. To test this hypothesis, we treated PC3 and DU145 cells with PA alone and in combination with compound C, an AMPK inhibitor. Our data showed that significant inhibition of phosphorylation of mTOR and S6 was observed in cells treated with PA alone. However, treatment with compound C reversed the reduction in phosphorylation in PA treated cells (Figure 7A).

Next we analyzed the role of AMPK in PA mediated autophagy. We observed no effect of compound C on the induction of LC3B in PA treated cells suggesting that induction of autophagy by PA is independent of AMPK (Figure 7B). We further assessed the role of AMPK in PA mediated apoptosis. Western blot analysis showed abrogation of PA induced cleavage of caspase 3 in compound C treated cells (Figure 7B). Flow cytometry was performed to quantify the number of cells undergoing apoptosis in PA treated cells exposed to compound C. Results showed that treatment with PA alone (20μM) resulted in 23.0% and 6.67% apoptosis in DU145 and PC3 cells, respectively but AMPK inhibition by compound C blocked PA induced apoptosis in both cell lines (0.41% and 0.3%) (Figure 7C; Sup. Figure 7A).

Combination studies of PA and metformin were performed to evaluate the effect on mTOR pathway and induction of autophagy and apoptosis. Western blot studies showed that treatment of PC3 and DU145 cells with either PA (20μM) or metformin (10mM) inhibited the phosphorylation of S6 but this inhibition was not observed in the combination treatment (Figure 7A). metformin reduced the induction of PA mediated cell death in PC3 cells, as assessed by a decrease in cleaved caspase 3 and LC3B expression in the combination group (Figure 7B & 7C; Sup. Figure 7A).

## DISCUSSION

The major findings of the current study are: i) PA, a novel compound isolated from the plant *Ficus microcarpa* possesses significant anti-proliferative activity against cancer cells; ii) PA is nontoxic to normal cells with selective toxicity to cancer cells; iii) PA binds to γ regulatory subunit with a high affinity and with low binding energy of −9.91 Kcal/mol; iv) PA is a potent activator of AMPK and targets AMPK to inhibit the viability of cancer cells; v) PA inhibits mTOR signaling and induces apoptosis in AMPK dependent manner; vi) PA induces autophagy independent of AMPK.

Our previous studies identified *Ficus microcarpa,* native to Pakistan, India, Taiwan and Australia as a rich source of bioactive compounds with potent antimicrobial and antioxidant properties [22]. Studies in albino wistar rats showed that the plant *Ficus microcarpa* possessed anti-diabetic activity [23]. Triterpenes isolated from the roots of *Ficus microcarpa* showed cytotoxicity to human nasopharyngeal and colorectal carcinoma cells [24]. In this study, we evaluated the activity of crude extract and fractions of *Ficus microcapra* on cancer cells and showed that the ethyl acetate fraction (FME) and the crude extract exerted potent anti-proliferative effect on melanoma and prostate cancer cells. We isolated two compounds, PA, a pentacyclic triterpenoid and FL, a flavonoid from the FME fraction (Figure 1). Cell viability studies demonstrated that PA, when compared to FL was more effective in inhibiting proliferation and viability of cancer cells. Cell cycle analysis of PA treated prostate cancer cells showed arrest in G0/G1 phase with accumulation of cell cycle inhibitors p21 and p27, associated with a decrease in Cdks and cyclins (Figure 2). This was accompanied with apoptosis in PA treated cells, as assessed by cleavage of PARP and Caspase-3 (Figure 3). Time course studies indicated PA treatment resulted in autophagy concomitant with induction of apoptosis (Figure 6B). Strikingly, relative to cancer cells, normal human epithelial cells were resistant to PA-mediated growth repression suggesting its potential use as a growth inhibitory agent against prostate cancer (Figure 1D).

One of the central regulators of cellular and organismal metabolism in eukaryotes is AMPK, with critical roles in growth, survival and cell polarity [8]. AMPK is a functional protein which is activated by number of stresses including glucose deprivation, ischemia, hypoxia and oxidative stress [25–27]. A recent interest in the role of AMPK in common chronic diseases, such as diabetes and cancer, has intensified studies of its regulation in cellular and animal models. Identification of new agents that can specifically target AMPK-dependent pathways has recently emerged as a field of intense and competitive studies. Our preliminary studies on PA indicated that PA possessed significant anti-hyperglycemic activity (unpublished data). Since several anti-hyperglycemic compounds are known modulators of AMPK activity, we examined AMPK as a molecular target for the observed PA-induced effects. Here, we provide evidence that PA is a novel potent activator of AMPK. Activation of AMPK requires phosphorylation of the kinase at the Thr^172^ residue by upstream regulators such as LKB1 and CaMKKβ [28]. Our studies in prostate cancer cells showed that PA stimulated AMPK activity independent of LKB1, and increased activity was observed both in LKB1 null DU145 and LKB1 positive PC3 cells, post PA treatment (Figure 4). A similar increase in AMPK activity was noted in PA treated hepatocytes (unpublished data) validating its role as an AMK activating agent.

*In silico* modeling, employed to determine potential interactions between PA and AMPK indicated regulatory γ subunit of AMPK as the preferred binding site for PA. The two polar ends of PA bind to γ subunit in a shape complementary manner (Figure 5). Labeling studies, using the reactive AMP analogue 8-azido-[(32)P]AMP have indicated that the γ subunit may participate directly in the binding of AMP within the complex [29]. Binding sites of PA was compared to the AMP binding sites and it was found that PA does not compete with AMP for binding (Figure 5Ac). Metformin is also reported to bind to γ subunit of AMPK, however unlike AMP or ZMP, metformin is not a direct allosteric AMPK activator [30]. Our data suggest that binding of PA to γ subunit of AMPK results in allosteric activation of the kinase, however further studies are warranted to determine the mechanism through which PA interacts with AMPK and induces the phosphorylation and activation of AMPK.

Although AMPK signaling was originally characterized as a tumor-suppressive signaling pathway, emerging evidence suggests that AMPK has divergent effects, and its role as an oncogenic regulator or tumor suppressor is subject to many variables including tissue model system [31]. mTOR, a downstream targets of AMPK, functions as an intracellular nutrient sensor to regulate protein synthesis and cellular growth and block catabolic processes such as autophagy at the post-translational and transcriptional levels [32]. Our studies show that PA negatively regulated mTOR signaling in prostate cancer cells in an AMPK dependent manner (Figure 6 & 7). Reduced AMPK activity has been reported to potentiate cancer cell growth through inhibition of the tumor suppressor tuberous sclerosis complex 2, known to suppress mTORC1 signaling, thereby augmenting the oncogenic stimulus for protein synthesis responsible for uncontrolled tumor cell growth [33].

PA induced autophagy in PC3 and NB26 but not in DU145 prostate cancer cells (Figure 6 & 7). Ouyang et al have shown that the autophagy pathway is genetically impaired in DU145 cells with several critical autophagy-related proteins, such as ATG5 and ATG12–ATG5 conjugates, being absent in this cell type [34]. The role of autophagy in cancer is controversial and still not completely clarified: it has been described as a double-edged sword because it is involved in both cell survival and tumor suppression, depending on cell type, stage of tumor development, nature of the stressor and genetic context [35]. Autophagy induced by PA seems to be protective as we observed less cell death in PC3 and NB26 as compared to DU145 (Figure 3). However, long term studies in animal models with or without the use of an autophagy inhibitor will delineate the role of PA induced autophagy in prostate cancer. Interestingly our data indicate that PA-induced autophagy is independent of AMPK, suggesting involvement of other pathways that are the subject of our ongoing studies in *in vitro* and *in vivo* models (Sup. Figure 7).

An important finding from our study is that AMPK activation is necessary and sufficient for PA-mediated suppression of tumor cell growth. These results are also in agreement with findings which suggest that the role of AMPK in cancer research is beyond its regulation of metabolic activity [12, 36, 37]. Comparative studies of PA with the widely used AMPK activator metformin suggest different mechanism of actions of these compounds (Figure 4 & 7). We found that metformin in combination with PA reduced apoptosis in prostate cancer cells which was more remarkable in PC3 cells. Since, metformin inhibited PA induced AMPK activity, it is therefore not surprising that PA-induced apoptosis, dependent on AMPK, is compromised by metformin.

Taken together, our studies show that PA is a novel compound, the potential pharmacological activities of which have hitherto remained unexplored. It has both anticancer and antidiabetic potential. The current study demonstrates a novel AMPK-mediated pathway that regulates cancer cell viability and growth in PA-treated prostate cancer cells and identifies AMPK as a potent therapeutic target of PA. Moreover, our results provide biochemical and molecular underpinning for direct targeting of AMPK in prostate cancer therapy given its potential to induce cell death in prostate cancer cells. Evaluation of the activity of PA in other preclinical models of cancer is mandatory for its translation as an effective agent against prostate cancer in clinical settings.

## MATERIALS AND METHODS

### Materials

Antibodies against cdk6, cdk2, cdk4, cyclin B1, cyclin D1, cyclin D2, cyclin E2, p27, p21, PARP, Bcl-2, Bax, Bcl-XL, p-AMPK, AMPK, p-ACC, ACC, p-mTOR, mTOR, p-S6, S6, p62, LC3AB and caspase-3 were purchased from Cell Signaling Technology (Beverly, MA). Anti-mouse and anti-rabbit secondary antibody horseradish per-oxidase conjugate was obtained from Amersham Pharmacia Life Sciences. The Bio-Rad DC Protein Assay Kit was purchased from Bio-Rad, CA. Novex precast Tris-Glycine gels were obtained from Invitrogen. The APO-DIRECT™ was purchased from BD biosciences. AMPK kinase activity was assay was performed by AMPK kinase activity kit which was purchased from CycLex Co., Ltd. (Cat#CY-1182).

### Cell culture

The following human non-tumorigenic (RWPE-1 & NHEK) and tumorigenic (DU145, CW22Rν1, PC3, NB26, and A375) cell lines were obtained from ATCC (Manassas, VA): cells. All cell lines were maintained as per the recommendations of ATCC.

### Cell viability assay

The effect of different treatments on cell viability was determined by 3-[4,5-dimethylthiazol-2-yl]-2,5-diphenyl tetrazoliumbromide (MTT). The absorbance was measured at 560 nm on a microplate reader (Bio-TEK Instruments).

### Clonogenic assay

The effect of treatment on clonogenic survival of prostate cancer cells was determined using colony formation assay. Prostate cancer cells (PC3, DU145 and NB26) were treated with PA (20 & 40μM). Following treatment, the cells were re-plated in triplicate on a 6-well tissue culture plate with 5000 cells/well and cultured in 5% CO2 at 37°C for 8 days with growth media being replaced with/without PA every 2 days. The cells were then stained with 0.5% crystal violet (methanol: H_2_O; 1:1) and pictures were taken.

### Cell cycle analysis/Apoptosis by flowcytometry

Prostate cancer cells treated with PA (20 & 40μM:24h), trypsinized and fixed in 1% Paraformaldehyde:1XPBS and washed thrice with PBS and centrifuged. The pellet was suspended in chilled 70% ethanol and stored overnight. The cells were labeled with FITC and propidium iodide using the Apo-Direct Kit (BD Pharmagen, CA) as per manufacturer's protocol. Analysis was performed with a FACScan (Becton Dickinson, NJ). About 10,000 events per sample were collected and the DNA histograms were analyzed with ModFitLT software (Verily Software House, ME).

### AMPK activity assay

AMPK activity assay of PA-treated cells was performed as per manufacturer's protocol. Absorbance was measured at 450 nm.

### *In silico* molecular dynamics study

Ligand docking studies were performed with Autodock4 (Scripps Institute). The model used for docking was the AMPK structure with ligands in the PDB entry 4CFF (DOI: 10.1038/ncomms4017, Nature Communications). α is cyan, β is green, γ is magenta and the ligands are gold. The α site is defined as the kinase binding site in the kinase domain of α. The β site is at the interface of the n-terminal α domain and the carbohydrate binding module (CBM) in β. There are three AMP binding domains in the allosteric regulator domain γ. Docking was performed with the docking box sizes large enough to include the binding sites. PA was modeled in Sybyl (Certara Co., www.tripos.com).

### Protein extraction and western blot analysis

Prostate cancer cells (PC3, DU145 and NB26) were cultured in 6 mm petri plates (2 × 10^5^ / plate). Cells were treated with 20 μM and 40μM PA for 24h. Following treatment, cells were washed with cold PBS (pH 7.4) after aspiration of media. Ice-cold lysis buffer was added lysis buffer. The composition of lysis buffer was 50 mM Tris–HCl, 150 mM NaCl, 1 mM ethyleneglycol-bis(aminoethylether)-tetraacetic acid, 1 mM ethylenediaminetetraacetic acid, 20 mM NaF, 100 mM Na_3_VO_4_, 0.5% NP-40, 1% Triton X-100, 1 mM phenylmethylsulfonyl fluoride, pH 7.4 with freshly added protease inhibitor cocktail (Protease Inhibitor Cocktail Set III, Calbiochem, La Jolla, CA). Plates were placed on ice for 30 min. The cells were scraped with the scraper and the lysate was collected in eppendorf tube and passed through needle of the syringe to break up the cell aggregates. The lysate was cleared by centrifugation at 14000*g* for 30 min at 4°C and the supernatant (whole-cell lysate) was used or immediately stored at −80°C. For western blotting 4-12% poly acrylamide gels were used to resolve 30μg of protein, transferred on to a nitrocellulose membrane, probed with appropriate monoclonal primary antibodies, and detected by chemiluminescence autoradiography after incubation with specific secondary antibodies.

### Statistical analysis

All statistical analysis was carried out with GraphPad prism (San Diego, CA) and p-values <0.05 were considered significant.

## SUPPLEMENTARY FIGURES


