# Supplementary material for: The pentacyclic triterpenoid, plectranthoic acid, a novel activator of AMPK induces apoptotic death in prostate cancer cells
Source: Oncotarget. 2015 Dec 16;7(4):3819–31. doi: 10.18632/oncotarget.6625 (PMC4826172; doi:10.18632/oncotarget.6625)
Supplement: Supplementary file 1 [file oncotarget-07-3819-s001.pdf]

## SUPPLEMENTARY FIGURES

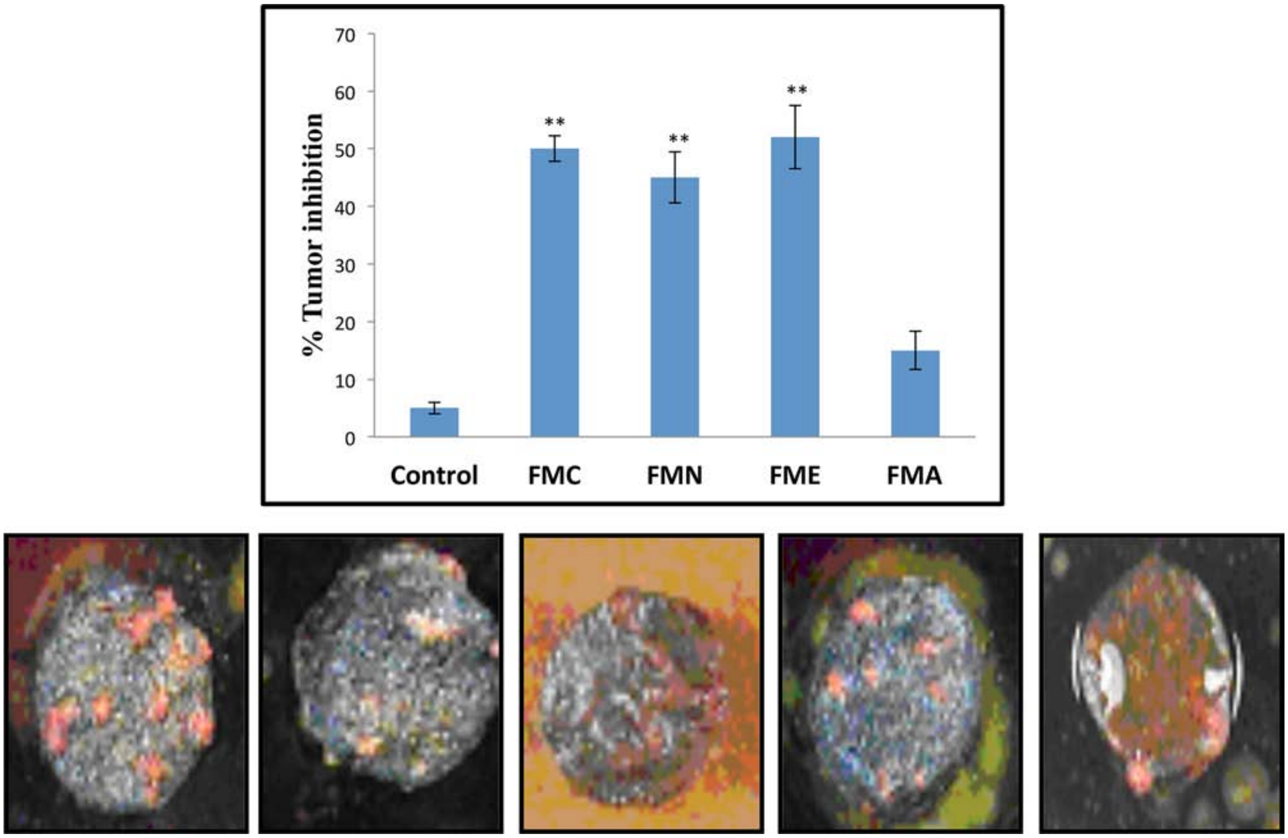


**Supplementary Figure S1: Potato disc antitumor assay showing tumor inhibition by *Ficus microcarpa* crude extract (FMC) and its fractions (FMN, FME and EMA).**

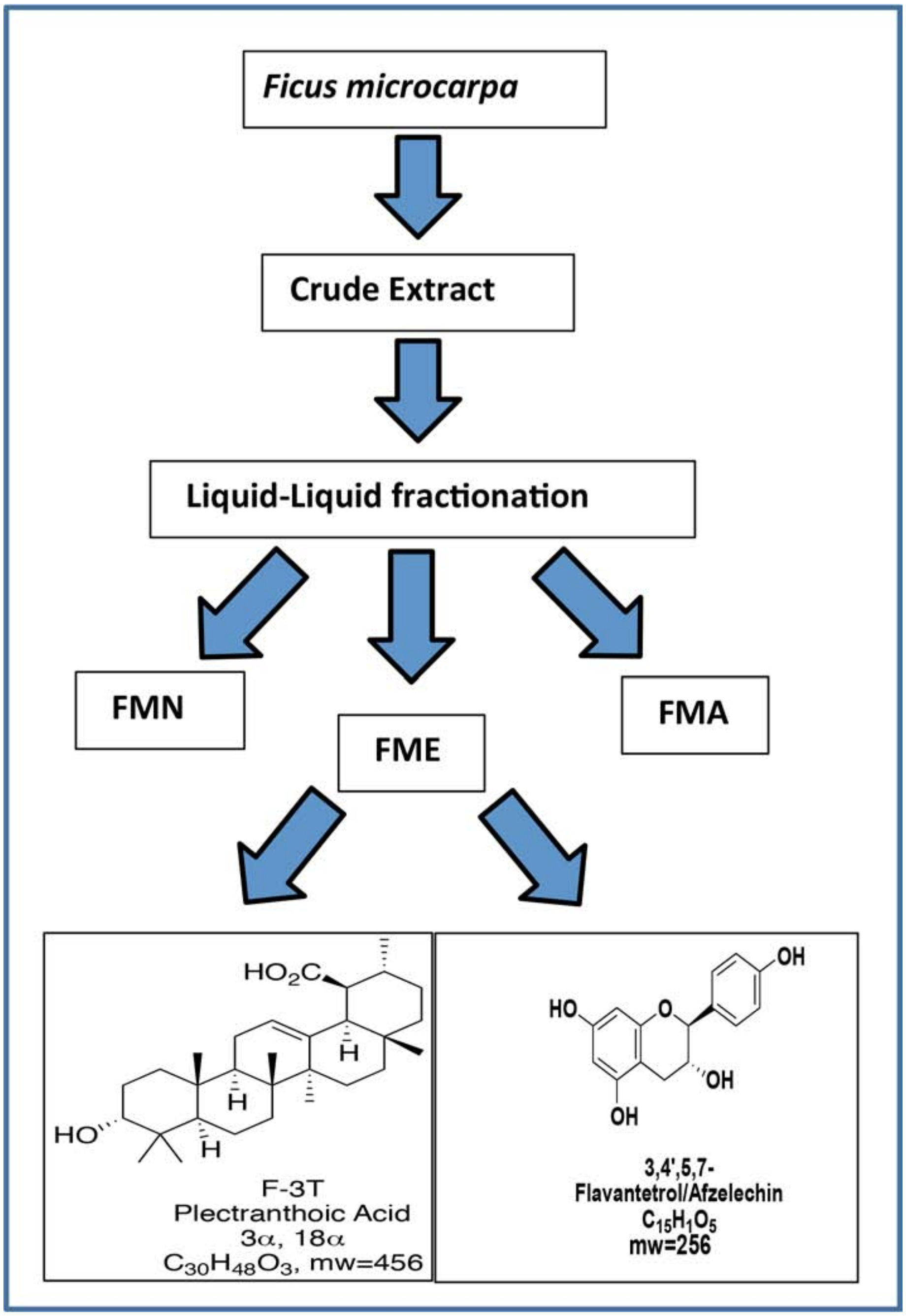


**Supplementary Figure S2: Flow chart for isolation of PA and FL from *Ficus microcarpa*.** Crude extract was separated into 3 fractions (FMN, FME and EMA) by liquid-liquid fractionation and both compounds were extracted from ethyl acetate fraction (FME).

A

| | DU145 (IC50: μM) | PC3 (IC50: μM) | CWRV1 (IC50: μM) | NB26 (IC50: μM) | A375 (IC50: μM) |
|---|---|---|---|---|---|
| FMC | 67 | 96 | 94 | 97.3 | 100 |
| FMN | 70 | 100.3 | 97 | 89.8 | 116 |
| FME | 70.4 | 89 | 94.1 | 88.3 | 108 |
| FMA | 101 | 152.3 | 112.9 | 72 | 146.1 |

B

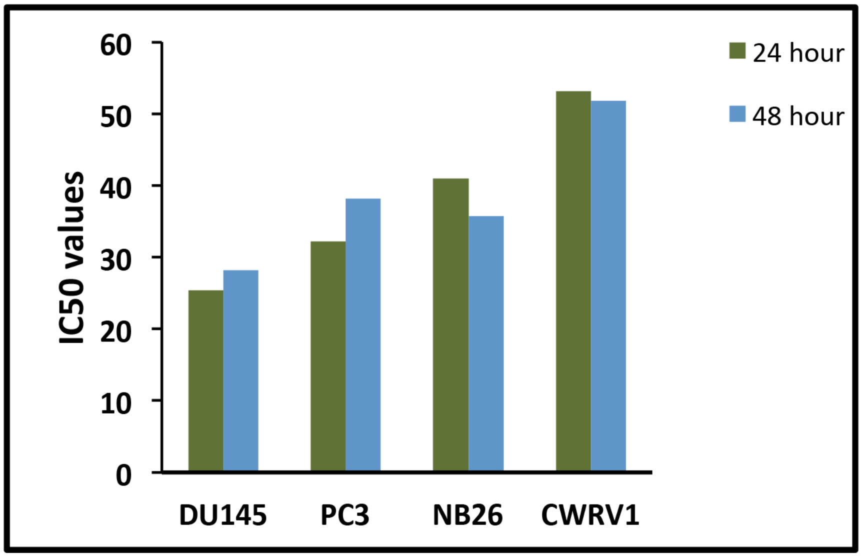


**Supplementary Figure S3: Effects of crude extract and fractions of *Ficus microcarpa* on the viability of melanoma and prostate cancer cells. A.** Cells were treated with FMC, FMN, FME and FMC at the indicated concentrations for 24h, and cell viability was assessed by MTT assays $IC_{50}$ values are tabulated. **B. Time course analysis of the effect of PA on prostate cancer cells:** DU145, PC3, NB26 and CWRV1 were incubated with PA for 24 and 48h, cell viability was assessed by MTT, and $IC_{50}$ values were calculated and plotted.

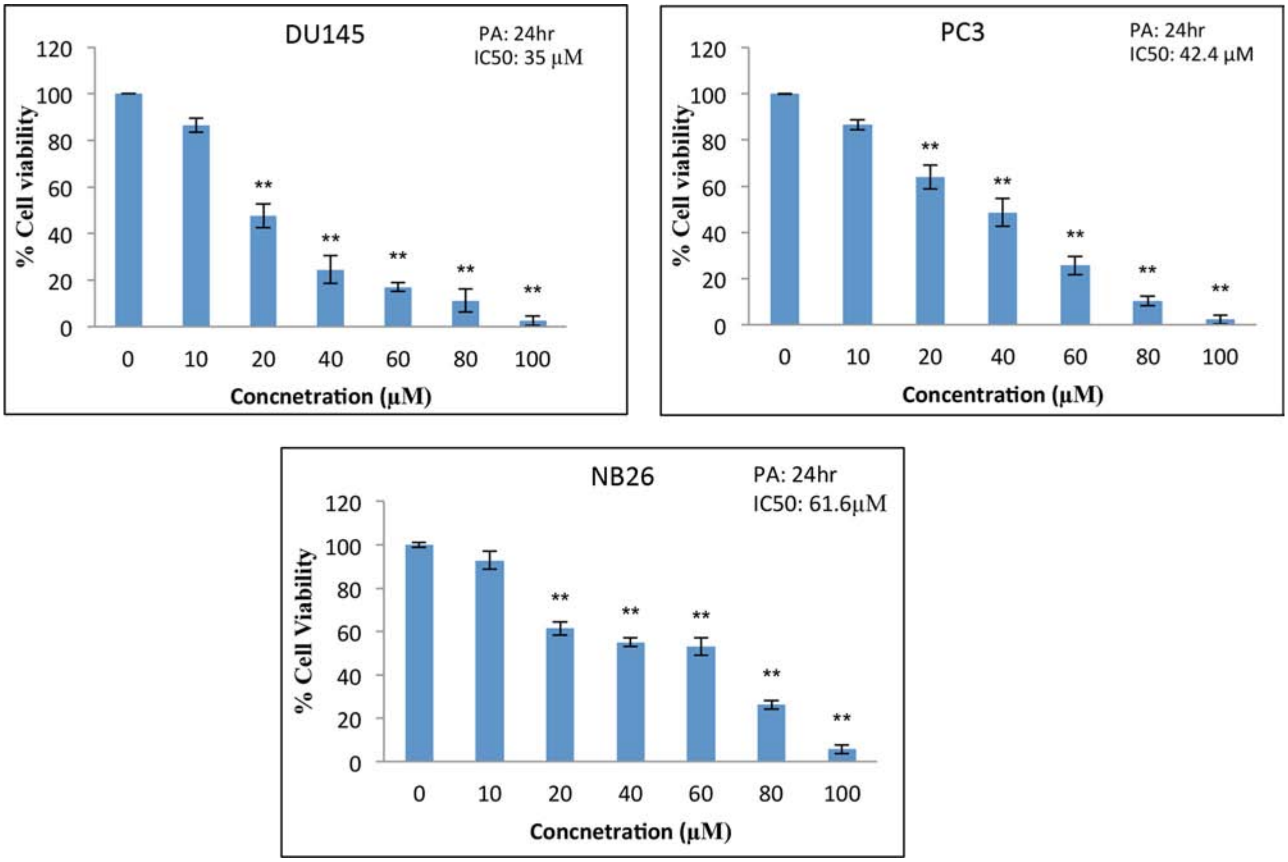


**Supplementary Figure S4: Effects of PA on the viability of prostate cancer cells.** Cells were treated with PA at the indicated concentrations in for 24h, and cell viability was assessed by BrDU assay (Cell Signaling), as per manufacturer's protocol.

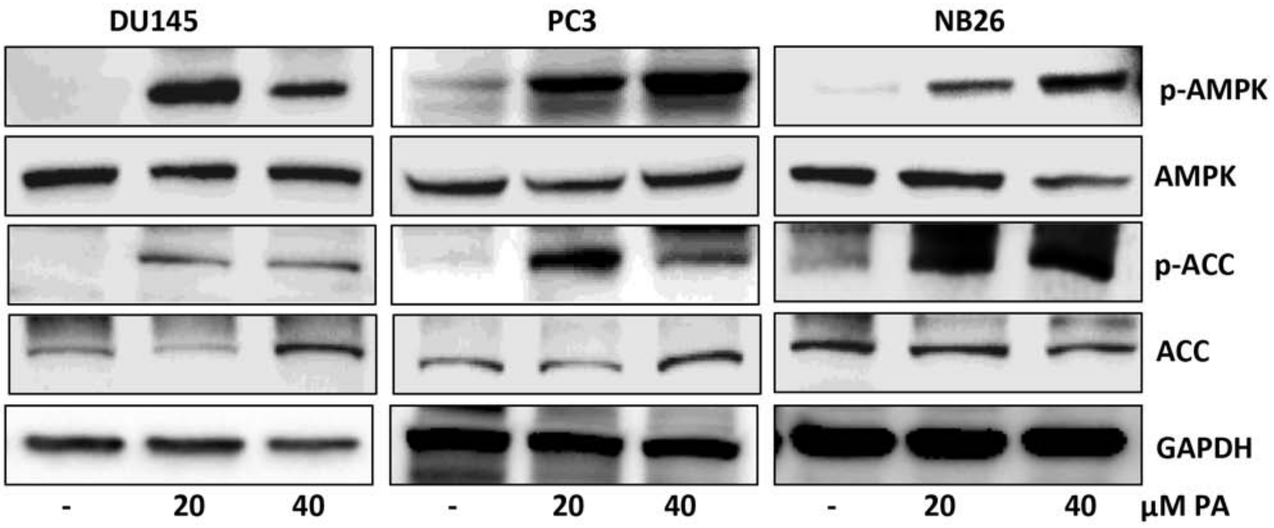


**Supplementary Figure S5: Effects of PA on AMPK signaling.** Whole cell lysates of PC3, NB26 and DU145 cells with/without PA (20–40µM:24h) treatment were subjected to SDS-polyacrylamide for expression of p-AMPK, AMPK, p-ACC and ACC. Equal loading was confirmed by reprobing with GAPDH. The immunoblots shown are representative of three independent experiments with similar results.

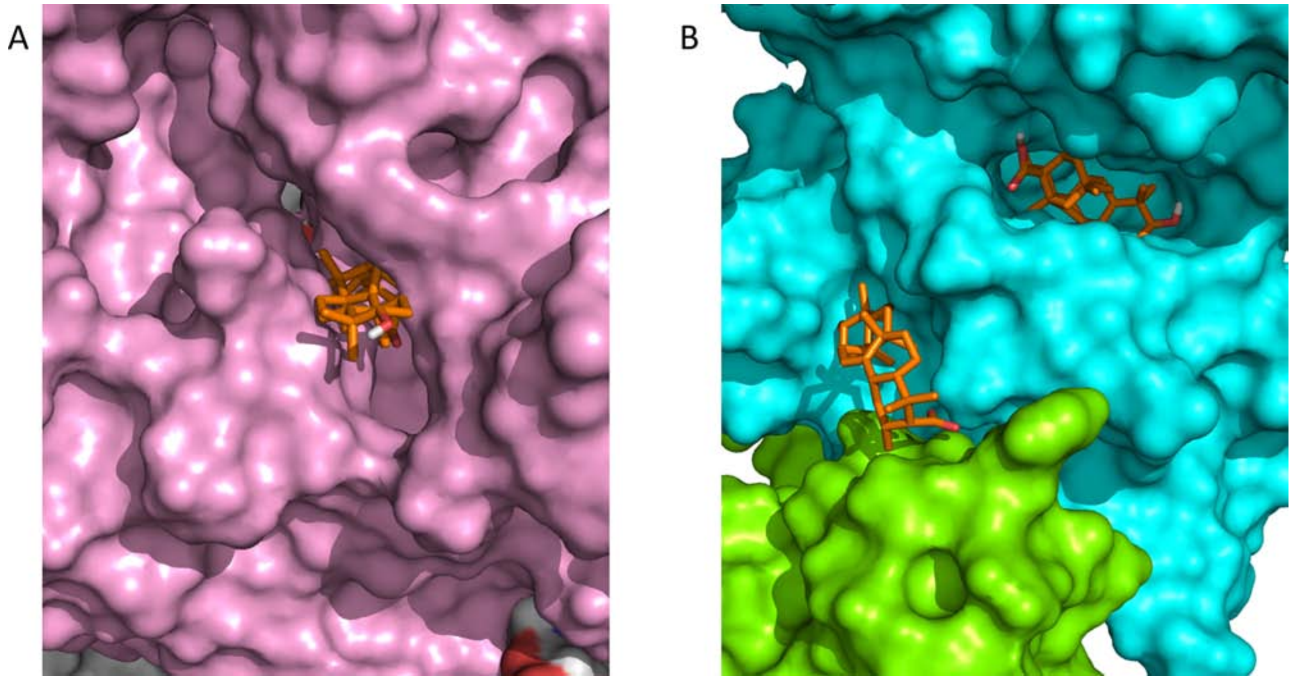


**Supplementary Figure S6: Binding of PA with γ, α and β subunits.** PA docked to AMPK by autodoc4. The model used was the AMPK structure with ligands in the PDB entry 4CFF (DOI: 10.1038/ncomms4017, Nature Communications). **A.** Surface pocket shows PA binding at γ subunit. **B.** Surface pocket shows PA binding at α and β subunits.

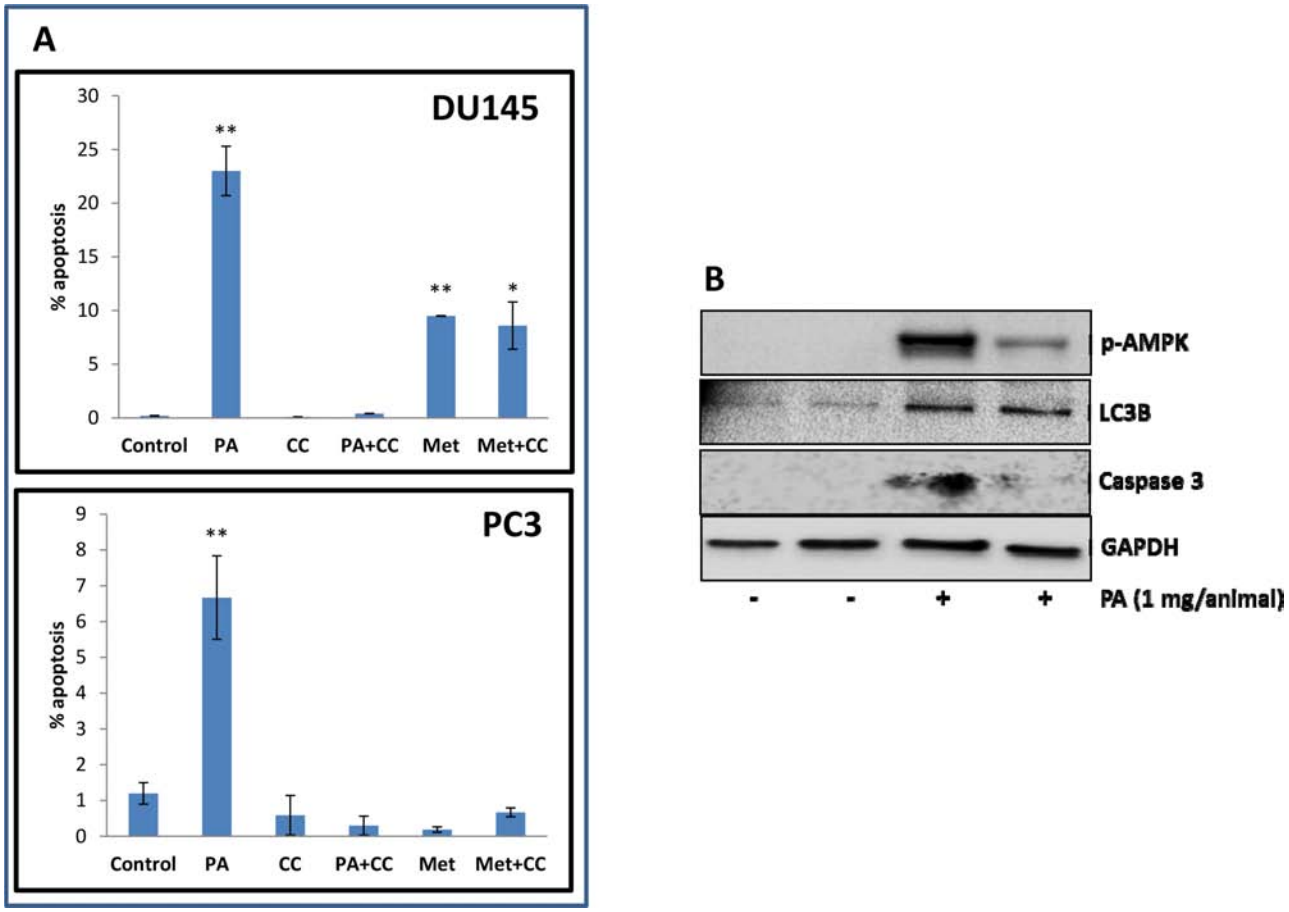


**Supplementary Figure S7: A. PA induces apoptosis in an AMPK dependent manner.** Cells were labeled with FITC and analyzed by flow cytometry. Percentage of apoptotic cells with different treatments are plotted in the graph. The results are expressed as normalized average ± S.D. of three independent experiments. **B.** Whole cell lysates of *CWR*22Rv1 xenografts implanted in athymic nude mice, treated with/without PA were subjected to SDS-polyacrylamide gel electrophoresis. Equal loading was confirmed by reprobing with GAPDH. The immunoblots shown are representative of three independent experiments with similar results.
